# Improving Adhesion of UHMWPE with Epoxy Matrix by Reactive Ion Etching of UHMWPE Using Ar–O_2_ Plasma and the Effects of Plasma on Adhesion at the Micro- and Macroscale

**DOI:** 10.1021/acsami.5c10473

**Published:** 2025-10-09

**Authors:** Usman Sikander, Mark K. Hazzard, Ian Hamerton, Michael R. Wisnom

**Affiliations:** a Bristol Composites Institute, School of Civil, Aerospace and Design Engineering, Faculty of Science and Engineering, Queen’s Building, University Walk, Bristol BS8 1TR, United Kingdom; b DSM Materials Science Centre, Urmonderbaan 22, 6167 RD Geleen, The Netherlands

**Keywords:** UHMWPE, plasma treatment, interface, adhesion, microbond

## Abstract

Ultrahigh molecular weight polyethylene (UHMWPE) fibers offer an excellent range of mechanical properties, but their applications in composites are limited due to their inert surface, which limits matrix wetting. This study employs reactive ion etching (RIE), using Ar–O_2_ gases to significantly improve the adhesion of UHMWPE fibers (with no surface finish) and tapes with epoxy at the micro- and macrolevels. Various oxygen-bearing functionalities are observed on the surface of the fiber after plasma treatment, confirmed by FTIR. These functional groups improve links between the unsized fiber and Prime20 LV epoxy resin. Consequently, the apparent interfacial shear strength (τ_IFSS_), measured by microbond testing, increases by 143%, 171%, and 181% as a result of plasma exposure for 10, 60, and 300 s, respectively, compared to untreated fibers. However, the frictional stress (τ_f_) in the postdebonded region of the microbond curve remains constant and independent of plasma exposure. Short-beam shear testing of interleaved composite laminates shows a 63% increase in the ILSS, along with a change of failure mode from interfacial failure to defibrillation of the tape itself after plasma treatment, mitigating a key limitation of using this material in structural applications.

## Graphical abstract

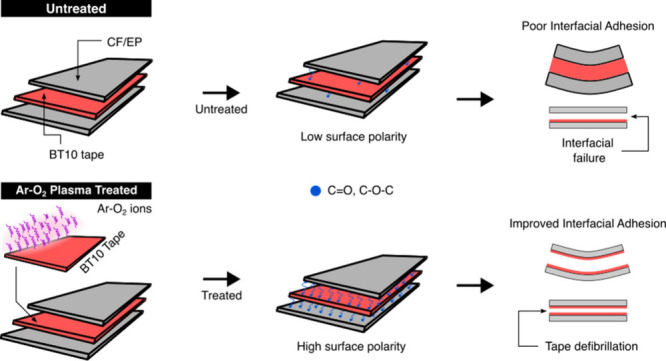

## 1 Introduction

Ultrahigh molecular weight polyethylene (UHMWPE) fibers have been widely adopted for applications such as ballistic armor protection,^1^ due to their high strength and energy-absorption capability while being lightweight.^2–4^ The fibers are either solution-grown or gel-spun^5,6^ and drawn, resulting in 95% molecular orientation in the drawing direction with a degree of crystallinity of 97.5%.^7^ The UHMWPE fibers have a relatively smooth surface and hierarchical structure, where the noncircular cross-section fiber of typically between 10 and 30 μm diameter consists of fibrils on both the micro- and nanoscale.^8^

Embedded commonly in thermoplastic matrices, various other applications^9^ of these fibers include but are not limited to ballistic helmets, vests, and frag-knits to protect the user from threats such as high-velocity ballistic projectiles. Studies have also been performed for use in structural applications through hybridizing with carbon epoxy composites.^10^ Recently this has shown excellent impact and energy-absorbing potential;^11^ however, the structural properties of a woven hybrid compared with a pure carbon-epoxy composite were negatively affected. Tensile and flexural strength was shown to reduce with increasing UHMWPE fiber content.^12^ It is hypothesized that the inert chemical nature and the tendency of the fiber to fibrillate can reduce off-axis properties, particularly when considering loads that induce shear. Because of this, we have investigated the effects of ion etching of UHMWPE using Ar–O_2_ plasma to improve the adhesion of UHMWPE with an epoxy matrix at the micro- and macroscale.

The fibrillar structure of UHMWPE is held together by weak van der Waals forces^15^ and tie molecules, with an estimated interfibrillar adhesive energy of 0.47 J m^–2^.^16^ The hierarchical structure of these fibers consists of a “shish-kebab” structure,^13^ where “shish” refers to thermodynamically stable extended chains and “kebab” refers to the lamellar structure, as shown in Figure 1. Owing to their molecular structure, these fibers have low surface energy and thus do not form strong interfacial bonds with thermosetting matrices.^10,17,18^ Thus, the fiber-reinforced polymer composites (FRPs) employing UHMWPE have low interfacial shear strength^19^ resulting in poor mechanical properties such as delamination and premature failure of the composite.^20^ While a low interfacial shear strength gives improved protection in ballistic protection systems,^21^ the use of UHMWPE fiber is severely limited in structural applications, due to the low interfacial and interlaminar shear strength in FRPs.^22^

**Figure 1:**
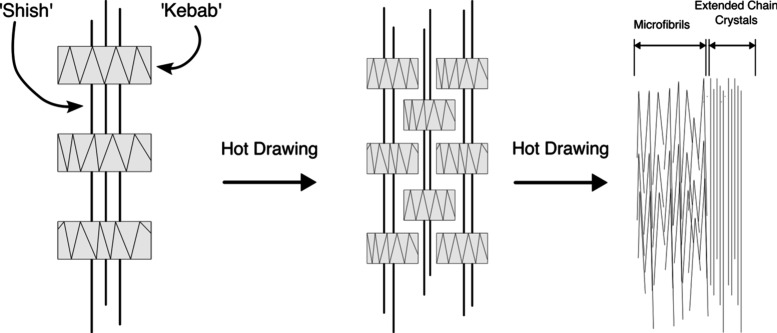
“Shish-kebab” structure of UHMWPE fibers, representing the alignment of fibrils toward the drawing.^13,14^

The interfacial strength of UHMWPE-epoxy composites was improved by various surface modification techniques. These modifications are brought by various routes such as coatings, wet routes (oxidizing agents), and dry routes (plasma treatment).^23^ The modifications improve the physicochemical interaction between the fiber and the matrices, i.e., the addition of functional groups and increased surface roughness leading to increased mechanical interlocking and hence improved adhesion.^23–25^

For instance, it has been shown that the interfacial shear strength (IFSS) of neat UHMWPE-epoxy increased from 1.06 to 3.03 MPa when the UHMWPE fibers were coated with polypyrrole (PPy).^26^ When the fibers were pretreated with plasma treatment, the IFSS increased to 10.05 MPa, and this increase was attributed to the formation of hydrogen bonds at the interface. A two-step coating process also showed promising results^27^ where polydopamine (pDA) was coated on the UHMWPE fibers, followed by grafting using ethylene glycol diglycidyl ether (EDGE). Increases of 13.2% and 21.5% were observed in the pull-out force for pDA-UHMWPE and pDA-EDGE-UHMWPE systems, respectively, when embedded in epoxy resin, compared to the neat fiber.

The benefits of coatings have also been explored for carbon and glass fibers where coatings with pDA and polynorepinephrine (pNE) have been shown to increase IFSS by 37% and 29% for carbon fibers and 27% and 18% for glass fibers, respectively.^25^ Similarly, the pDA coatings for UHMWPE, modified with −COOH functionalized multiwalled carbon nanotubes (MWCNTs),^2^ showed a 19.6% increase in mode-I fracture energy for pDA-coated UHMWPE and 42.5% increase in pDA+CNT modified UHMWPE fibers when embedded in an ELIUM 188 matrix.

Various forms of wet chemical routes have also been employed to improve the adhesion between the fiber and the matrix. For example, it was shown that when the high-performance polyethylene (HP-PE) hybrid UD laminates were treated with chromic acid, an increase in ILSS was observed.^10^ The treatment also changed the failure behavior; i.e., the untreated samples showed longitudinal shear between the HP-PE and carbon fibers along with excessive debonding, whereas the treated samples showed a brittle failure of the HP-PE fibers.

Cracking of fibers was also observed when the UHMWPE fibers were treated with potassium permanganate and nitric acid and used as a UHMWPE-natural rubber system.^28^ In this case, the longitudinal cracks along the fiber axis were thought to have increased the surface area of the fibers. The treatment increased the oxygen functionality on the fibers’ surface, and both reasons were attributed to an increased adhesion between the two phases.

Strong etching agents like chromic acid, potassium permanganate, and hydrogen peroxide may increase oxygen content on the surface of the fibers but are known to reduce the tensile strength and failure strain of the fibers. For example, a 50% reduction in tensile strength and a 45% reduction in failure strain of UHMWPE fibers have been reported, when these fibers are treated with chromic acid.^28^ Such treatments also lead to oxidation of the surface of UHMWPE, forming oxygen-rich weak boundary layers (WBL), which hinder the adhesion process.^29^

Plasma treatments, on the other hand, are less hostile to the surface of fibers but still provide functionality to the fiber surface. For example, the argon (Ar) plasma has been reported to reduce the strength only by 10%,^29^ which is significantly less than a 20–50% reduction observed when using a strong oxidizing agent such as chromic acid.^30^ The detrimental effects of plasma, i.e., the extent of reduction in tensile strength as a result of plasma exposure, are beyond the scope of this paper and not included in the study.

The present study explores the tangible benefits of the reactive ion etching (RIE) process to effect significant improvements in the adhesion between UHMWPE fibers and epoxy resin. The plasma process parameters were chosen based on a review of previous studies,^24,31–35^ with a notable emphasis on low plasma exposure times in this paper to avoid excessive fiber damage.

RIE is a low-pressure process (10^–1^ to 10^–3^ Torr), compared to the conventional plasma treatment (10^2^ to 10^–1^ Torr) and offers a unique combination of plasma etching and ion milling, resulting in a precise and directional etch compared to the conventional plasma treatment.^36^

Specifically, this study shows how RIE can alter the surface roughness of the fibers but also identifies the role of surface roughness in controlling the adhesion properties at the surface. Such effects are studied on both the micro- and the macroscale in this work, along with monitoring changes in surface chemistry and their effects on adhesion between UHMWPE-epoxy systems.

Although various plasma parameters (i.e., plasma gases, process parameters, types of plasmas) have been used in several studies in the past three decades on a variety of systems including UHMWPE-epoxy, there exists a gap in understanding the relationship between reactive ion etching, surface roughness, and its effects on adhesion at the micro- and macroscale. The focus of this study, on unmodified fibers, is particularly important as the spin-finish applied to other grades of DyneemaⓇ may significantly affect the adhesion characteristics of the fibers, thus obscuring the proposed effects of plasma treatment. However, by focusing on Trevo 90 fibers, a grade of DyneemaⓇ that contains no surface finish and a material that rarely features in scientific studies, it has been possible to isolate and quantify the effects of RIE on altering the surface roughness of the fibers and also identify the role of surface roughness in controlling the adhesion properties at the surface. Such effects are studied on both the micro- and the macroscale in this work, along with monitoring changes in surface chemistry and their effects on adhesion between UHMWPE-epoxy systems.

## 2 Experimental Section

### 2.1 Materials

The DyneemaⓇ Trevo90 fibers and BT10 tapes,^37^ with no spin-finish, were supplied by DSM N.V., The Netherlands (ρ = 0.975 g cm^–2^, nominal diameter = 12–21 μm, tape areal weight = 90 g m^–2^). The BT10 tape was composed of unidirectional UHMWPE fibers, prepared by hot-pressing calendaring of UHMWPE powder, to form 20 μm thick highly aligned polymer sheet.^38^ The tape did not contain any matrix material.

The epoxy system used for investigation was Prime 20LV resin (≈652 cP, 1.123 g cm^–3^) with extra slow hardener (13–15 cP, 0.931 g cm^–3^) from Gurit, Newport, United Kingdom. The single fiber microbond samples were held and prepared in a mold, which was cast using Polycraft GP-3481-F silicone rubber, supplied by MB Fibreglass. The microbond samples were held in the acrylic end tabs by Ultra-Light-Weld-3193 UV curable resin supplied by Dymax Europe GmbH.

### 2.2 Reactive Ion Etching

Samples of BT10 tape were cut into 20 mm × 20 mm sections, whereas Trevo90 fibers (length <50 mm) were cut from the yarn. Both these sample types were placed on a water-cooled substrate electrode (dia = 280 mm, *h* = 105 mm) of RIE Plasma Pod Plus, manufactured by JLS Designs, Ltd. The plasma treatment was conducted using argon (Ar) and oxygen (O_2_) at 8 and 42 SCCM gas flow rates at a process pressure of 50 mTorr. The samples were exposed to plasma for 10, 60, and 300 s at 100 W RF plasma power. Only one side of the specimen was exposed to plasma in this study.

### 2.3 Microbond Testing

Trevo90 single filaments were extracted from the yarn for microbond samples and were prepared by holding the fibers in acrylic end tabs and securing them in place using the Ultra-Light-Weld-3193 UV curable adhesive. The UV-curable resin was cured in the resin dams of acrylic end tabs using CoolLED pE-100 UV probe (λ = 365 nm, 10–20 s). The Prime 20LV resin was mixed with the extra slow hardener in a ratio of 100:26 using an overhead stirrer for 15 min. The resin/hardener mixture droplets were then applied to the UHMWPE fibers using the tip of H-glass fiber. The geometry of the microbond samples, i.e., fiber diameter (*d*_f_), embedment length (*l*_e_), and droplet diameter (*d*_drop_), was measured using Zeiss Axio upright microscope at ×10 magnification.

A large number of microbond samples were prepared; however, only a few samples were used to determine the τ_IFSS_, as shown in Table 2. The sample acceptance/rejection was based on three criteria, where samples were rejected if (i) resin droplets (*d*_drop_) were greater than 450 μm and (ii) droplets had improper geometry and (iii) Chauvenet’s criterion eq 1, where IFSS values outside a threshold of 0.5 of a two-sided probability (0.674σ in this study) were treated as an outlier and removed from the estimation.

1 0.5>(P(X,μ(N),σ(N))−1)N

where *P*, *X*, μ, and σ represent probability (50%), the sample mean, the population mean, standard deviation, and the total sample size, respectively.

All samples were tested using a 50 μm microvise width gap, displaced by 2 mm at a speed of 0.0016 mm s^–1^ and a contact force of 0.1 g. The samples were tested using the LEX820 module of the single fiber tester supplied by Diastron Ltd. The force vs displacement response of samples was recorded, noting the maximum force (*F*_max_) at which debonding occurred. The *F*_max_ value was then plotted against embedment length, and using the linear least-squares optimization method,^39^ the apparent interfacial shear strength (τ_IFSS_) was determined according to eq 2, using the radius of the fiber (*r*_f_).

2 $$\mathrm{gradient}=\tau_{\mathrm{IFSS}}2{\pi r}_{f}$$

where gradient is the slope of linear regression line, in *F*_max_ vs *l*_e_ plot. The frictional stress (τ_f_) in the pull-out region was computed according to eq 3:^40^

3 $$\tau_{f}=\frac{F_{\mathrm{deb}}}{2{\pi r}_{f}l_{e}}$$

where *F*_deb_ was the postdebonding force determined from experimental results, taken as the first value after *F*_max_, *r*_f_ was the fiber radius, and *l*_e_ was the embedded length of the droplet. The debonding force (*F*_deb_) was defined as the immediate datum after *F*_max_, assuming that the frictional force remained constant throughout the pull-out region after debonding.^40^

### 2.4 Fourier Transform Infrared (FTIR) Spectroscopy

Fourier transform infrared (FTIR) spectroscopy was performed on Trevo90 single fibers and BT10 tapes in ATR mode using a PerkinElmer 100 spectrometer. The spectra were obtained in the range of 4000–600 cm^–1^, at a scan resolution of 4 cm^–1^ and scan accumulation of 16 scans per sample.

### 2.5 Scanning Electron Microscopy

The morphology and energy-dispersive X-ray spectroscopy (EDS) of the pristine and failed microbond samples were studied using a Joel JSM-IT300 scanning electron microscope (SEM), using an X-max 80 mm^2^ Silicon Drift Detector and Aztec 2.3 software, both supplied by Oxford Instruments. The high-resolution images of the pristine and fractured sites of the samples were acquired under a high vacuum at accelerating voltages of 15.0 keV in secondary electron (SE) and backscattered electron (BSE) modes.

### 2.6 High-Speed Atomic Force Microscopy

The surface morphology of untreated and treated UHMWPE fibers was studied using the Bristol Nano Dynamics Ltd. Gen 4, contact mode high-speed atomic force microscope (HS-AFM). The instrument scanned 2000 lines per second at 2 frames per second, using a Bruker MSNL cantilever C tip with a tip radius of 5 nm. Several frames in an area of 4.7 μm^2^ on the fiber’s surface were captured. No flattening was performed at the time of the data acquisition. However, to account for the bowing effect, the raw data were postprocessed to remove the curvature using open-source software Gwyddion,^41^ and both the raw and the flat data sets were compared. The flattening was performed using the median plane, where the real radius was kept at 0.132 μm and a pixel radius of 20 pixels. After leveling, the rows on the surface were aligned using the first-degree polynomial method, followed by subtracting the mean plane from the data. The data were reset to 0 μm on the *Z* scale, and the 2D/3D images were compared and used to measure the surface roughness parameters.

### 2.7 Composites Samples Preparation

The unidirectional (UD) laminates were fabricated using SE 84LV prepreg (intermediate modulus carbon fiber, *V*_f_ = 55.5%), supplied by Gurit (United Kingdom), employing BT10 tape in the midsection as an interleaf material. The composite laminate was fabricated using hand-layup and consisted of 12 plies at 0° with dimensions 120 mm × 120 mm. Vacuuming bagging was performed, as shown in Figure 2. A caul plate was added to the top to ensure that the samples had a smooth finish, i.e., to comply with ASTM D2344 ^42^ guidelines for the short beam strength test. The laminates were cured as per the manufacturer’s recommended cure cycle: (i) heat up to 87 °C at 1 °C min^–1^, (ii) dwell for 12 h at 87 °C for 6 h, and (iii) cool to room temperature.

**Figure 2:**
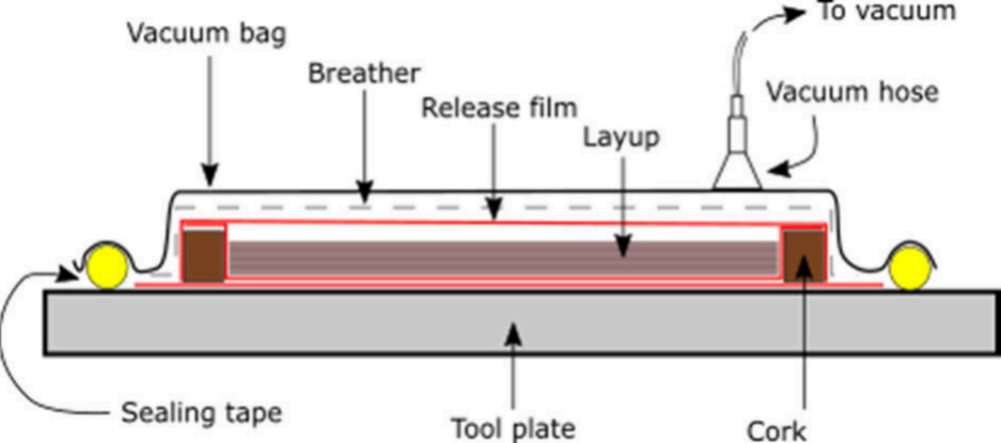
Schematic representation of the vacuum bagging process for CF-epoxy and CF-DyneemaⓇ-epoxy laminates.

A vacuum was maintained at −1 bar throughout the curing cycle. Three types of laminates were produced and tested, i.e., (i) all carbon-epoxy laminates (L1), (ii) carbon-epoxy laminates with untreated BT10 tape in midsection (L2), and (iii) carbon-epoxy laminates with plasma-treated BT10 tape in midsection (L3). The plasma treatment on interleaved samples was conducted using Ar–O_2_ (1:5) plasma at 100 W power, 50 mTorr process pressure, where only one side of the BT10 tape was exposed to plasma for 10 s, and the gas flow rate was 8 SCCM and 42 SCCM for Ar and O_2_, respectively.

### 2.8 Short Beam Shear Testing

The samples for short beam shear (SBS) testing were cut in dimensions of 22.0 mm × 10.0 mm × 1.85 mm using water jet cutting and machining. Guidelines in ASTM D2334 ^42^ governed the specimen dimensions. The SBS testing was performed at a span-to-depth (*S*/*D*) of 5.0,and at a crosshead speed of 1 mm min^–1^.

## 3 Results and Discussion

### 3.1 Surface Characterization of UHMWPE Fibers

#### 3.1.1 Morphology

The surface morphology of UHMWPE fibers in the pristine and plasma-treated states can be seen in Figure 3a–d. The fibrillar nature of the fibers was confirmed with the occasional presence of kinks, which could have been due to the bending of fibers during the handling stage. The fibers in pristine condition displayed a smooth and shiny surface, as shown in Figure 3a. However, after plasma treatment, the surface appeared to be altered. The plasma treated fibers showed presence of débris, which could be attributed to the removal of material (fiber fragments) and degradation of surface, because of the interaction between plasma and the fibers.

**Figure 3:**
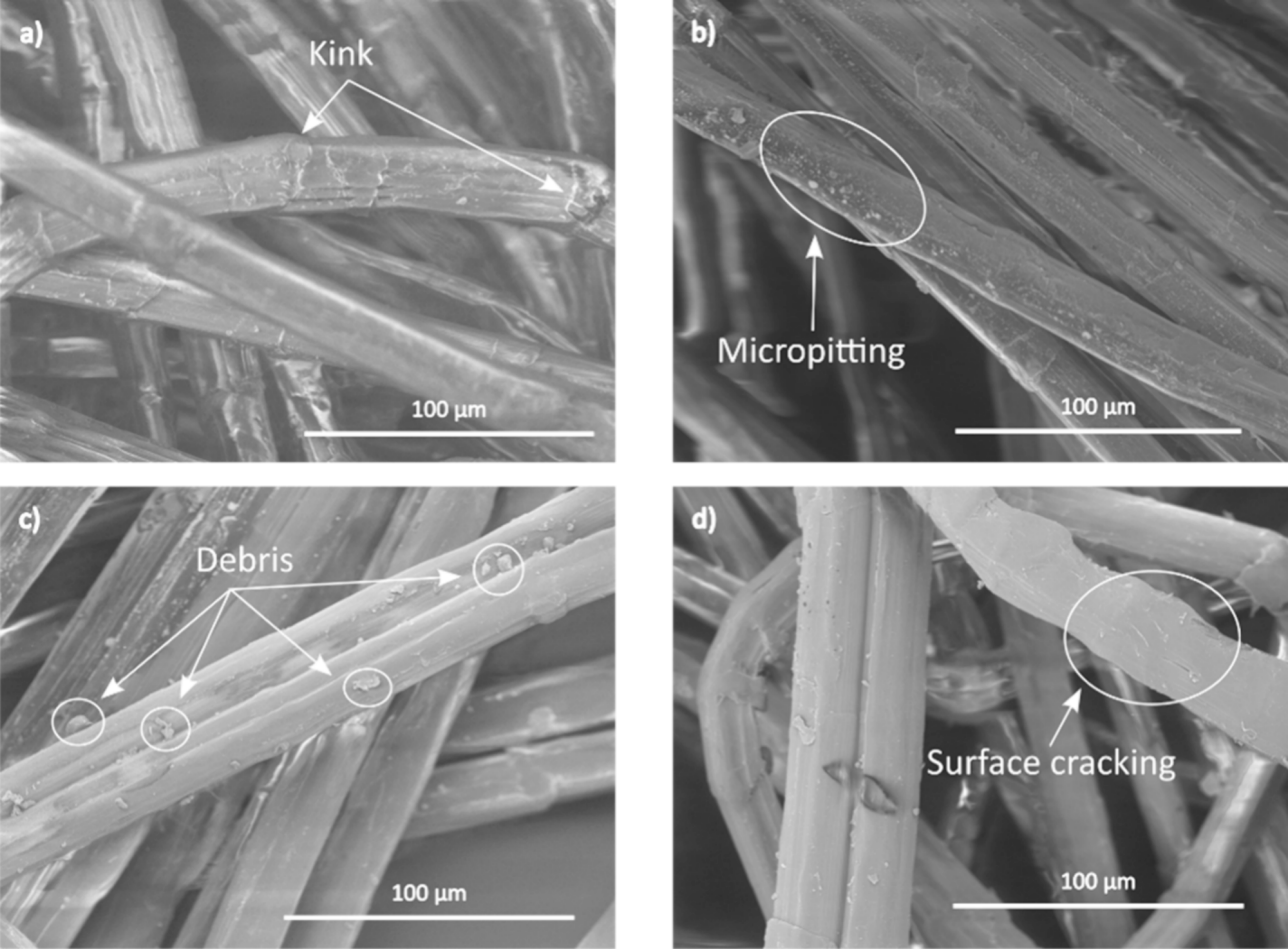
SEM of Trevo90 fibers in (a) untreated state and plasma-treated state at a plasma exposure time of (b) 10 s, (c) 60 s, and (d) 300 s. The samples were exposed to Ar–O_2_ plasma at 100 RF power and 50 mTorr process pressure with gas flow rates of 42 and 8 SCCM for Ar and O_2_, respectively.

Specifically, the fibers were treated for 10 s (Figure 3b) showed micropitting on the surface,^40^ whereas the samples exposed to plasma for 60 s showed débris on the surface (Figure 3b). When exposed to plasma for 300 s, surface cracks, along the longitudinal axis of the fibers, were observed as shown in Figure 3d. The surface condition of the fibers, before and plasma exposure, is further elaborated in Figure S.1.

#### 3.1.2 Surface Roughness

The AFM data were acquired for Trevo90 fibers in an untreated and plasma-treated state, and the surface roughness parameter *S*_q_ along with the projected area (*A*_proj_) are tabulated in Table 1. The *S*_q_ parameter is the root-mean-square deviation of the surface which describes the amplitude of the surface.^43^ It is a three-dimensional extension of the two-dimensional *R*_q_ parameter and measures the deviation of the surface.

**Table 1:** Surface Roughness (*S*_q_) and Projected Area (*A*_proj_) of Untreated and Plasma-Treated Trevo90 Fibers

exposure (s)	data type (−)	*S*_q_ (nm)	*A*_proj_ (μm^2^)
0	raw	48.1 ± 20.6	19.9 ± 1.2
10	raw	53.8 ± 12.6	20.8 ± 0.6
60	raw	13.2 ± 6.2	20.8 ± 0.8
300	raw	27.0 ± 7.0	20.7 ± 0.5
0	flat	2.0 ± 0.6	19.7 ± 1.2
10	flat	2.4 ± 0.4	20.9 ± 0.6
60	flat	2.3 ± 0.3	21.0 ± 0.4
300	flat	2.1 ± 0.2	20.7 ± 0.5

A *t* test found that there was no significant difference in the surface roughness *S*_q_ values between the untreated and the samples treated for 10 s (*p* > 0.05). However, at a higher exposure time of 60 and 300 s, a significant difference in *S*_q_ was observed (*p* < 0.05). These observations were true for both the raw and flattened data. Thus, the surface roughness resembled that of untreated fiber following treatment with plasma for 10 s. However, from 10 s of treatment onward the roughness decreased appreciably at exposure times of 60 and 300 s. Therefore, the variation in the surface roughness parameter *S*_q_ did not vary linearly with increasing plasma exposure time, which was similar to observations made in.^44^

The flattened 3D rendered images of the fiber surface, in both the untreated and plasma-treated state, along with the line profiles in the longitudinal and transverse direction are shown in Figure 4a–e. The surface of the fiber was relatively smooth in an untreated state (Figure 4a), and roughening was observed when the samples were exposed to plasma. The samples exposed for 10 s (Figure 4b) showed surface metrology similar to the untreated fiber. However, the samples exposed to plasma for 60 s (Figure 4c) and 300 s (Figure 4d) showed a reduction in roughness and reorientation, respectively, relative to the untreated fiber.

**Figure 4:**
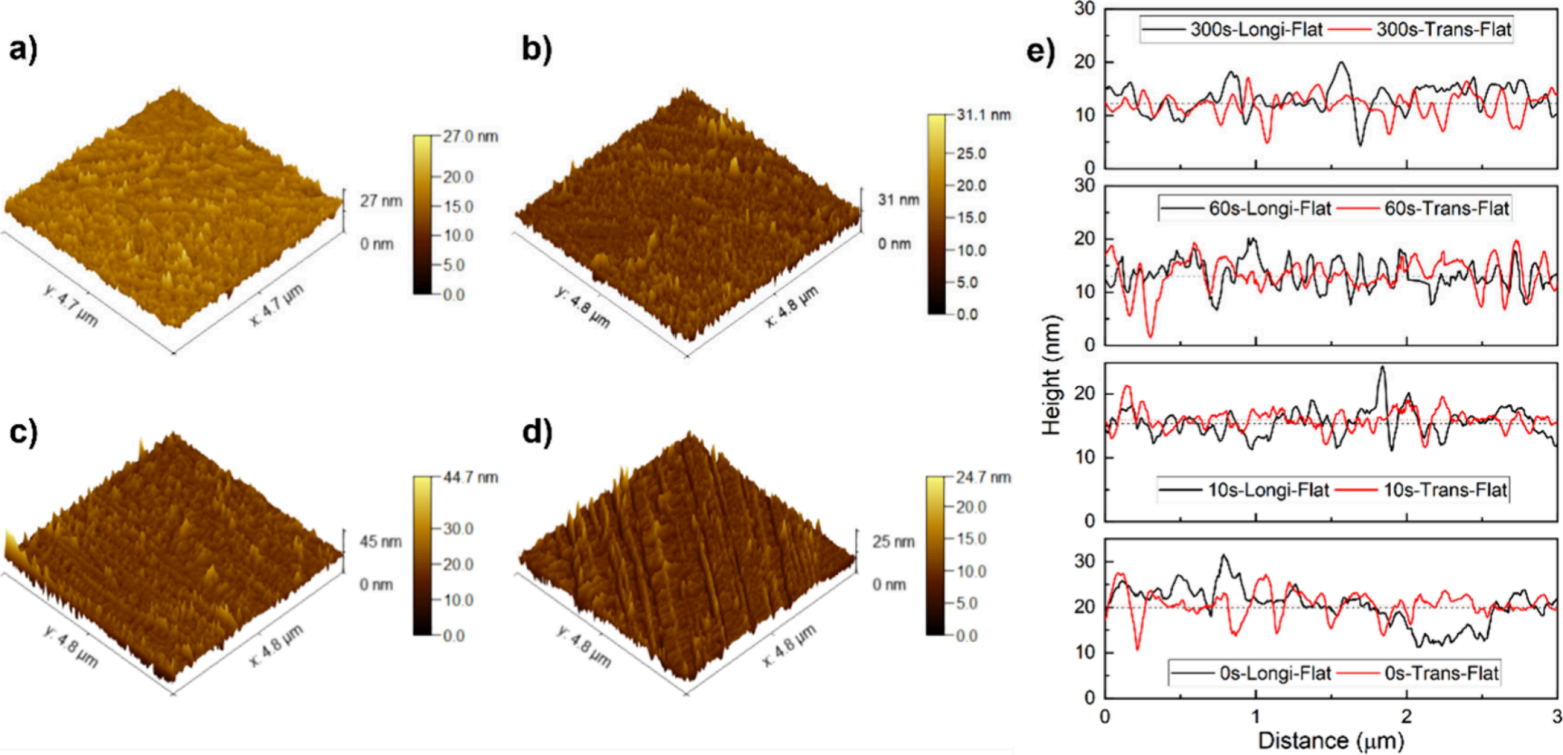
3D rendered images of the surface of (a) untreated and plasma-treated Trevo90 fibers, exposed to plasma for (b) 10 s, (c) 60 s, (d) 300 s, and (e) the corresponding line profiles in the longitudinal and transverse direction.

The decrease in surface roughness at higher exposure time was attributed to the ablation of the surface due to the continuous bombardment of higher energy particles.^45^ Such an interaction could have led to material removal and formation of channels in the case of samples exposed to plasma for 300 s, as shown in Figure 4d. Thus, at a lower exposure time of 10 s, the Ar–O_2_ plasma did not alter the surface roughness. At the higher exposure time of 300 s, surface reorientation could have been due to a preferential attack of oxygen on amorphous regions.^46^ Other surface roughness parameters such as *S*_a_, *S*_p_, *S*_v_, and *S*_*z*_ were also measured and are tabulated in Table S.1.

### 3.2 Surface Chemistry

#### 3.2.1 Fourier Transform Infrared Spectroscopy

The changes in surface chemistry were determined using FTIR spectroscopy, where the spectra of untreated (as) and plasma-treated samples are shown in Figure 5. The presence of hydroxyl functional groups (−OH) was observed at 3330 cm^–1^.^47,48^ The peaks observed at 2913 and 2847 cm^–1^ were attributed to asymmetric and symmetric vibrations of methylene functional groups (−CH_2_−).^49^ The peaks at 1472 and 1462 cm^–1^ were attributed to symmetric bending and rocking vibrations of methylene groups.^49^ The peaks at 730 and 716 cm^–1^ were associated with out-of-phase^47,50^ and in-phase vibrations of the methylene group.^22^

**Figure 5:**
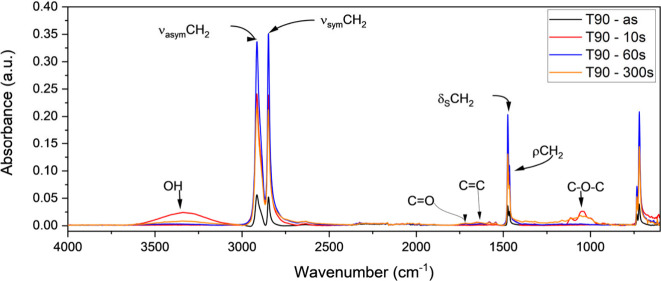
FTIR spectra of untreated and plasma-treated (PT) Trevo90 fibers, exposed to Ar–O_2_ plasma for 10, 60, and 300 s.

Various oxygen-bearing functional groups (e.g., C–O–C at 1035 cm^–1^) were observed in the spectra when the fibers were plasma-treated. The formation of C=C double bonds (at 1630 cm^–1^) after plasma treatment indicated that the σ bonds between carbon and hydrogen are broken, and thus unsaturated π bonds were formed between neighboring carbon atoms.

When UHMWPE fibers are exposed to plasma, the topmost layer of the fibers undergoes surface modification within a fraction of a second, along with modifications occurring at the subsurface layers, concurrently.^51^ A prolonged exposure changes the modification regime from functionalization to etching, which can result in the formation of a host of moieties, such as low molecular weight oxidized material (LMWOM), cross-linking on the surface of the fiber, formation of double bonds, etc.^51^ Consequently, unsaturation on the surface of fiber occurs; i.e., species with double bonds are formed. In the present study, the presence of such unsaturated bonds can be found as weak-medium bands in the region of 1600–1715 cm^–1^,^50^ associated with C=C or C=O groups.^52^

### 3.3 Effect of Plasma Treatment on IFSS

The droplets of Prime 20LV resin, cured on the pristine and plasma-treated Trevo90 fibers, were displaced using a microvise. The force vs. displacement curves of the microbond test are shown in Figure 6a, and the linear-elastic portions of the curves are shown in Figure 6b. Failed microbond specimens were also studied using SEM to determine the characteristics of microbond specimens in postdebond condition. The droplet geometry and indentation features are presented in Figure S.5 in the Supporting Information. The presented data show representative curves for each of the sample types, and the analysis was performed only on the accepted samples. The total number of samples tested, accepted, and rejected, is presented in Table 2.

**Figure 6:**
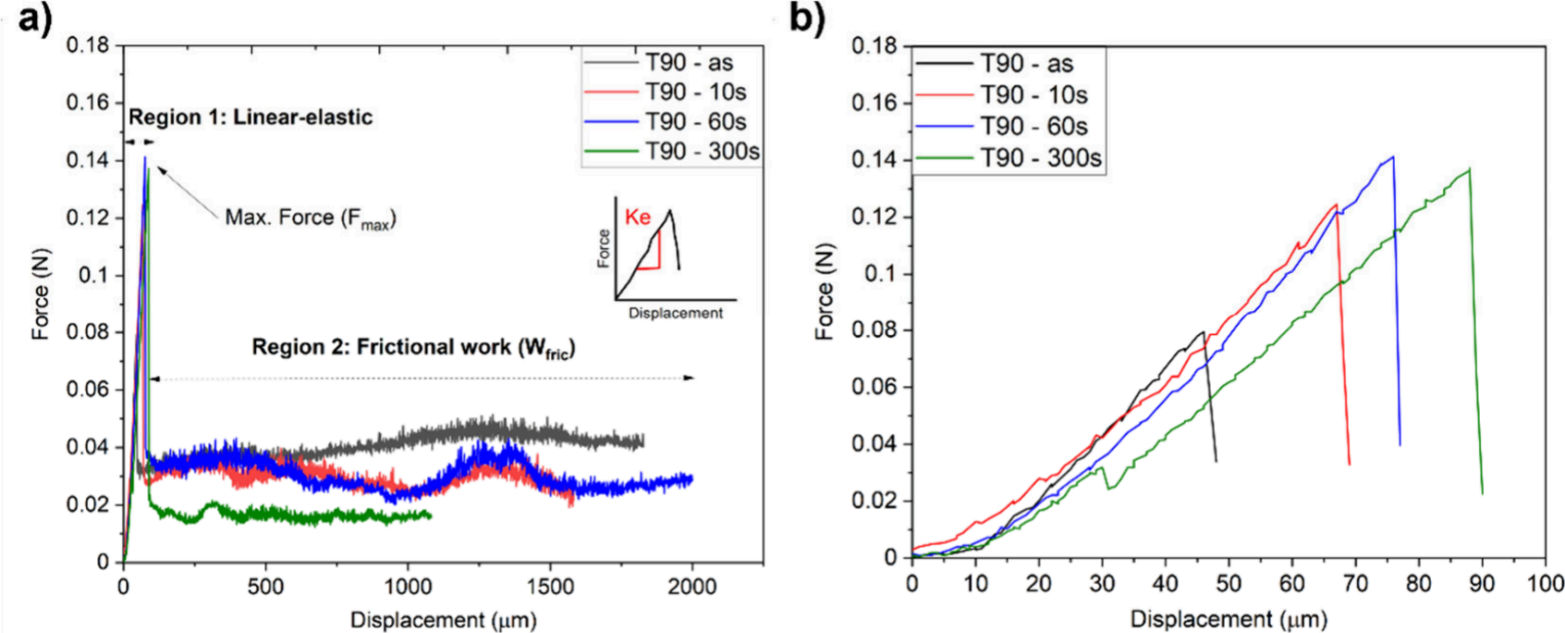
(a) Force vs displacement response of single fiber microbond test, (b) linear-elastic response of single fiber microbond tests showing a drop in force at maximum force (*F*_max_).

**Table 2:** Accepted vs. Rejected Samples for Microbond Testing

sample	total no. of samples	accepted	rejected	% accept
T90 – as	89	26	63	29
T90 – 10s	43	22	14	51
T90 – 60s	43	29	14	67
T90 – 300s	52	45	7	87

Two distinct regions, i.e., (i) elastic deformation and (ii) fiber pull-out, can be seen in Figure 6a. In region i the load is taken up by the fibers from 0 N to *F*_max_, where debonding occurs at *F*_max_. Once debonded, the force rapidly drops, leading to the pull-out of the droplet in the region ii. An oscillating frictional force (*F*_fric_) was observed during the pull-out, representing the “stick and slip” mechanism.

The increase in force up until *F*_max_ and sudden drop followed by frictional pull-out resembled the case of shear debonding, as presented in.^53^ The stick and slip mechanism was also observed in the double cantilever beam (DCB) tests conducted for UHMWPE-pDA and UHMWPE-pDA-CNT systeMS^2^, where the oscillation of such variation was attributed to strong interfacial bonding between the fiber and the matrix. A similar response for carbon fibers embedded in amine-cured HY-914 epoxy resin was observed,^54^ where the force in the pull-out region was reported to be constant. However, unlike UHMWPE-pDA,^2^ the carbon/HY-914 study^54^ attributed the fluctuations to intermittent contact between fiber and droplet during the pull-out.

In the present case of Trevo90 fibers and Prime 20LV resin, the debonding force (*F*_max_) and hence the interfacial shear strength (τ_IFSS_) increased due to plasma treatment, but the frictional force (τ_f_) remained constant, as shown in Table 3. The stick and slip mechanism was also observed for the Trevo90/Prime 20LV system (Figure 6a), and in some cases, a “hump” in the force was observed during the frictional pull-out of the fiber. Such a variation in *F*_fric_ was associated with changing fiber diameter along the length of the fiber. During the pull-out, increasing fiber diameter could have led to a higher contact; hence, a higher frictional force was observed locally at some points in this region.

**Table 3:** Comparison of Interfacial Shear (*τ*_IFSS_) and Frictional Stress (*τ*_f_) for Untreated and Plasma-Treated Trevo90

sample (−)	avg *F*_max_ (N)	avg *F*_deb_ (N)	avg *l*_e_ (mm)	avg *r*_f_ (mm)	avg τ_IFSS_ (MPa)	avg τ_f_ (MPa)
T90 – as	0.073 ± 0.03	0.040 ± 0.02	0.379 ± 0.10	0.013 ± 0.00	2.1 ± 0.40	1.2 ± 0.40
T90 – 10s	0.123 ± 0.06	0.036 ± 0.02	0.349 ± 0.07	0.012 ± 0.00	5.1 ± 1.10	1.4 ± 0.80
T90 – 60s	0.152 ± 0.05	0.041 ± 0.02	0.365 ± 0.08	0.012 ± 0.00	5.7 ± 1.00	1.5 ± 0.60
T90 – 300s	0.147 ± 0.06	0.028 ± 0.02	0.377 ± 0.09	0.011 ± 0.00	5.9 ± 0.50	1.0 ± 0.50

The interfacial shear strength was estimated using eq 2, where the values of *r*_f_ and the gradients were determined from experimental results. From Table 3, it can be seen that the untreated fibers showed significantly lower τ_IFSS_ values than the plasma-treated samples. Comparison of τ_IFSS_ of samples plasma-treated for 10 and 60 s showed statistically significantly different IFSS values. Similarly, the IFSS of samples treated for 10 and 300 s also showed significant differences. However, no significant difference in IFSS between 60 and 300 s of treatment was observed. Compared to untreated samples, plasma treatment increased the IFSS by 59%, 63%, and 64% when samples were exposed to Ar–O_2_ plasma for 10, 60, and 300 s, respectively.

It should be noted that the data set presented in Table 3 excludes the outliers, which were detected using (i) Chauvenet’s criterion,^55^ where outliers were removed using a two-sided probability of a suspected observation at a 50% threshold value, and (ii) inconsistent droplet geometry. In other words, apart from Chauvenet’s criterion, droplets larger than 450 μm in diameter were not used in the analysis. The large droplets also present the problem of incomplete cure due to diffusion of the hardener,^56^ thus reducing the mechanical properties of the resin cured on the single fibers.

It has been reported in the literature that the interaction of plasma with polymers is physiochemical in nature; i.e., such interaction leads to changes in surface chemistry and surface roughness of polymers. Such changes, and specifically the increase in surface roughness, are believed to increase the adhesion between the fiber and the matrix.^22,24,25,32,57,58^ However, in the present case, no change in surface roughness was observed at a lower exposure time of 10 s (Table 1), but τ_IFSS_ increased. Even for higher exposure times, the magnitude of τ_IFSS_ did not vary linearly with surface roughness parameter *S*_q_.

For example, the untreated samples showed a raw *S*_q_ value of 48.1 ± 20.6 nm, and after 10 s of exposure to Ar–O_2_ plasma, the roughness value was estimated to be 53.8 ± 12.6 nm, hence an insignificant difference (*p* > 0.05, Table 1). However, the τ_IFSS_ was estimated to be 2.1 ± 0.4 MPa for untreated and 5.1 ± 1.1 MPa for the samples treated for 10 s (Table 2). Similarly, the *S*_q_ values changed significantly at exposure times of 60 and 300 s, relative to untreated fiber. Although the change in τ_IFSS_ was small between 60 and 300 s of treatment, it was statistically significant. Therefore, it was inferred that the surface roughness did not affect the IFSS values and an increase in IFSS could be due to the addition of oxygen-bearing chemical species as a result of plasma treatment.

This was indeed the case. Plasma exposure at a lower level (10 s) does not change the surface roughness at a significant level; however, significant changes in IFSS (2.1 to 5.1 MPa for 0 and 10 s, respectively) were observed. A higher IFSS was attributed to an increased oxygen functionality after 10 s, which was confirmed through FTIR data (Figure 5 and Figure S.2), showing enhanced oxygen functionality across all examined wavenumber regions.

A higher peak values for C–O–C, C=O, and OH functional groups for 10 s, compared to 0 s, could have led to a higher chemical affinity for bonding between fiber and matrix, leading to an increased IFSS value. However, this level of plasma exposure may not have been sufficient to bring significant changes in surface roughness; thus *S*_q_ remains broadly unchanged (Table 1).

At a higher plasma exposure time of 60 and 300 s, the IFSS value plateaus (5.1 to 5.0 MPa, Table 3) while surface roughness decreases. This shows that while oxygen functional groups may be saturated at the surface of the fiber, surface roughness can decrease due to etching or ablative effects of plasma, without decreasing the adhesion at micro level. Thus, chemical functionality dominates the adhesion between the matrix and the fiber, and adhesion increases as a result of exposure.

The estimated values of frictional stress in the postdebonded region remained constant; i.e., no significant differences were found in the magnitude of τ_f_ when samples were treated for 10, 60, and 300 s of plasma exposure. Although there were differences in surface roughness observed, the maximum difference between different exposure times and an untreated fiber *S*_q_ was 0.4 nm for flattened data. In perspective, this is less than 0.01% of the smallest measured radius of the smallest filament (11 μm), and as such, no difference in frictional stress was observed.

### 3.4 EDS of Microbond Samples

EDS was performed on both untreated and plasma-treated samples after the droplets were pulled on the single fibers. This analysis aimed to determine any trace amounts of epoxy left at the initial droplet sites after the samples failed. The EDS spectra are shown in Figure 7a,b, and the initial droplet sites are presented in Figure S.4 in the Supporting Information.

**Figure 7:**
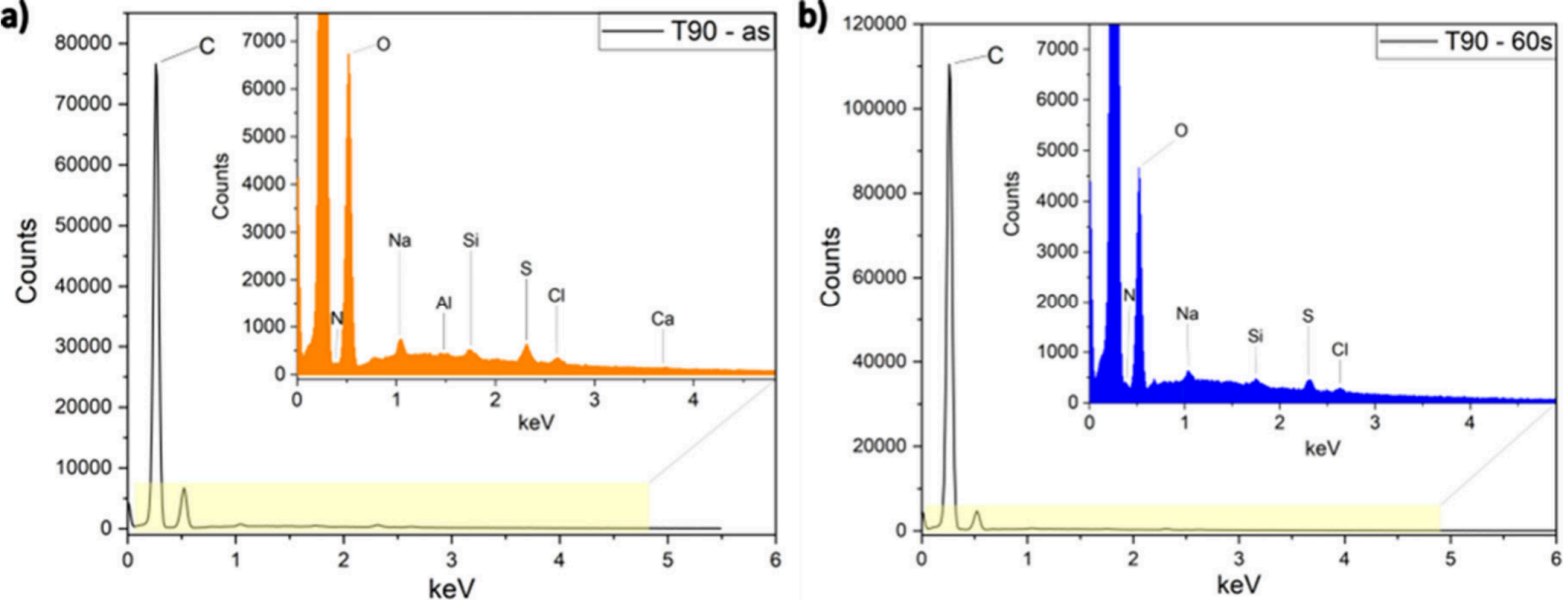
Spectra of (a) untreated and (b) plasma-treated Trevo90 fibers, exposed to plasma for 60 s.

As shown in Figure 7a, aside from carbon and oxygen, a range of chemical elements, i.e., N, Na, Ca, Cl, Cu, W, Si, and S, were observed on the surface of untreated T90 fibers. The presence of such chemical species is likely due to contamination during the manufacturing process through environment and/or tooling. However, once the plasma treatment is conducted, the surface of plasma-treated samples showed the absence of species such as W, Ca, and Cu, as shown in Figure 7b. The absence of these species after plasma treatment was associated with the “cleaning” aspect of plasma treatment.^15,29,33,51^

The elemental maps of both untreated and treated fibers were similar because carbon and oxygen were the primary constituents, and hence it was challenging to distinguish between the fiber and the matrix. However, the presence of oxygen on the surface could have been detected since the Prime 20LV resin used in this study had various polymeric fractions that contained oxygen-bearing species. The T90 fibers showed no residual epoxy at the initial droplet sites, before and after plasma treatment (Figure S.3).

### 3.5 Interlaminar Shear Strength on the BT10 Composites

The results of the short-beam shear test are presented in Figure 8, and the configurations of laminates L1, L2, and L3 are presented in section 2.8. From the force vs. displacement curves (Figure 8), two data points were observed: (i) the point of maximum load (*P*_m_), and (ii) the first point of load drop (*P*_drop_) for the interleaved specimen before reaching the maximum load. The *P*_drop_ data points were observed because of the configuration of the specimen.

**Figure 8:**
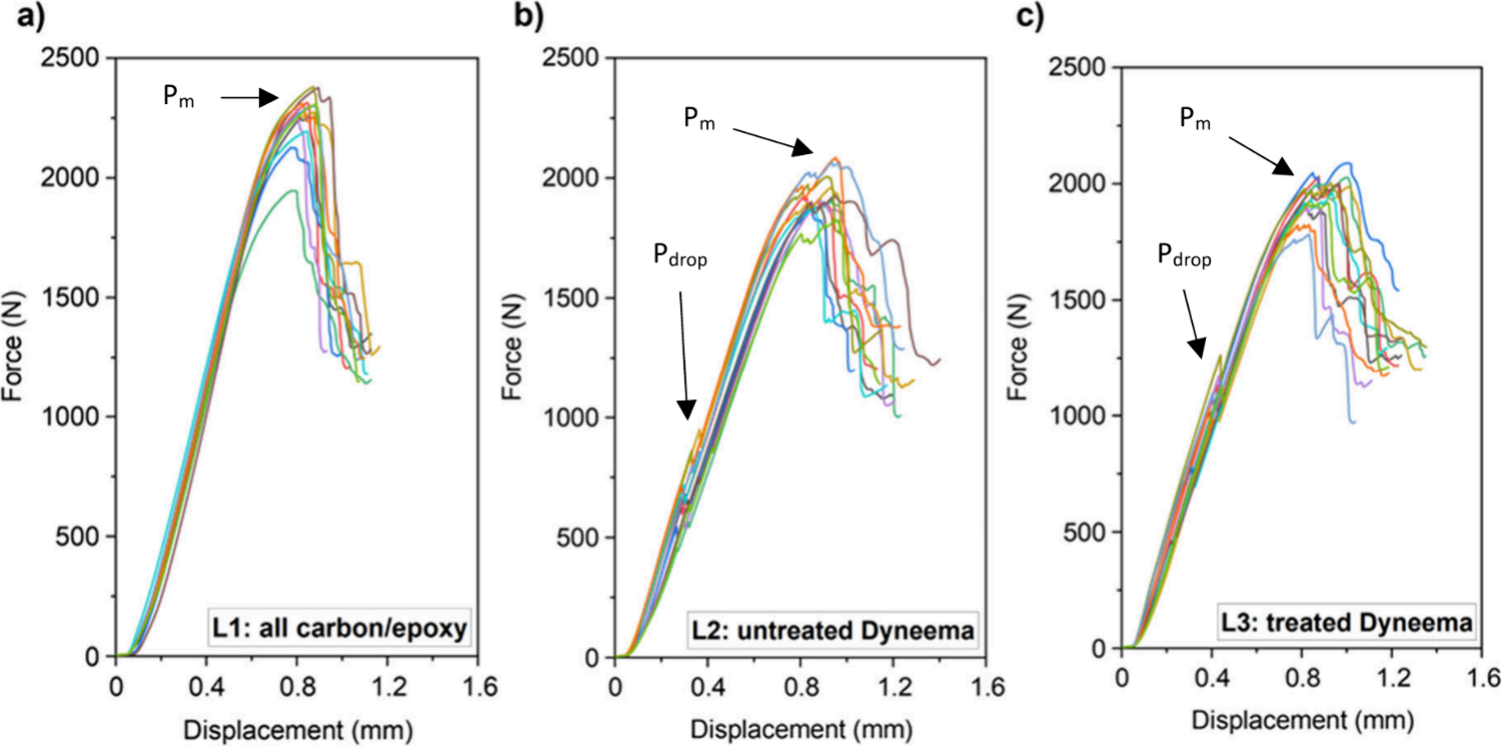
Force vs displacement curves of (a) untreated, all carbon-epoxy laminate (L1), (b) carbon-epoxy and untreated BT10 tape laminates (L2), and (c) carbon-epoxy and plasma-treated BT10 tape laminates (L3). The samples were exposed to Ar–O_2_ plasma for 10 s at 100 W RF power using Ar and O_2_ at flow rates of 8 SCCM and 42 SCCM, respectively, at a process pressure of 50 mTorr.

Short beam specimens normally fail with a load drop occurring at the maximum applied force due to interlaminar shear failure near the midplane, and the short-beam strength is usually calculated from the maximum load (*P*_max_), as per guidance provided in ASTM D2344.^42^ The ASTM D2344 serves as a guide and may not always measure the true shear strength due to the complexity of internal stresses and the possibility of a variety of failure modes. However, these specimens behaved differently with a load drop occurring much earlier. It would be easy to miss this, but it is very important as it corresponds to the failure between the DyneemaⓇ tape and the adjacent plies and occurs at consistent loads. Further, a *S*/*D* of 5 was chosen over 4, to promote interlaminar shear failure, as opposed to compressive failure at the loading nose and supports.^59^

Examples of conventional specimens can be seen in laminate L1, which did not employ the DyneemaⓇ interleaf, and therefore no load drop was observed, before reaching *P*_m_. However, for laminates L2 and L3 which employed DyneemaⓇ interleaves, a sizable load drop was observed before reaching the maximum loads.

These load drops were associated with the failure of the interface and the failure of DyneemaⓇ tape itself, for L2 and L3 laminates, respectively, and were found to occur at significantly different loads. Laminate L1 showed a maximum load (*P*_m_) of 2256.1 ± 120 N compared to 1938.8 ± 77.9 and 1954.7 N ± 88.7 N, for L2 and L3, respectively, but these are not what is of interest. The load drop (*P*_drop_) corresponding to DyneemaⓇ failure for the L2 and L3 laminate was substantially higher for the treated samples, increasing from 652.1 ± 124.7 N to 1066.3 ± 145.6 N. Thus, it can be seen that the plasma treatment greatly affected the magnitude of *P*_drop_.

The interleaved specimens were expected to show a load drop much lower than the *P*_m_ (as observed for all carbon/epoxy specimens) because of their low adhesion and shear strength, hence acting as a plane of weakness between the carbon-epoxy plies. Nevertheless, it was surprising to see that the samples with untreated (L2) and plasma-treated (L3) BT10 tape showed a significant load-carrying capacity up to the load drop, i.e., 26.0 ± 4.5 and 42.3 ± 6.2 MPa, respectively. Thus, plasma treatment changed the failure mode of samples interleaved with BT10 tape, which is further discussed in section 3.6.

Compared to the all carbon-epoxy laminate, the samples with DyneemaⓇ BT10 interleaf were able to carry load beyond the point of failure of the interface or the tape itself, as shown in Figure 8b,c. The short-beam shear strength (*F* ^SBS^) of all the laminates based on the first load drop is presented in Table 4 and the load drops are graphically shown and tabulated in Figure S.3 and Table S.3.

**Table 4:** Results of Short-Beam Shear Strength Test of All Carbon-Epoxy (L1), Carbon-Epoxy and Untreated BT10 (L2), and Carbon-Epoxy and Plasma-Treated BT10 (L3) Laminates

laminate (−)	*P*_drop_ (N)	avg *L* (mm)	avg *W* (mm)	avg *t*	*F* ^SBS^ (MPa)	CV (%)
L1		22.1 ± 0.0	10.0 ± 0.0	1.9 ± 0.06	88.1 ± 4.1	5.3
L2	652.1 ± 124.7	22.0 ± 0.1	10.0 ± 0.0	1.9 ± 0.1	26.0 ± 4.5	17.0
L3	1066.3 ± 145.6	22.0 ± 0.3	9.9 ± 0.0	1.9 ± 0.0	42.3 ± 6.2	15.0

The comparison of load-carrying capacity was made using the *P*_drop_ in the force vs displacement curves shown in Figure 8. Laminate L1 showed no load drop before reaching the maximum load (*P*_m_), unlike laminates L2 and L3 which showed load drops, marked as *P*_drop_, before the maximum load was reached. Therefore, the load-carrying capacity for laminate L1 was based on *P*_m_ whereas that for L2 and L3 was based on *P*_drop_ because failure had occurred even though it was able to continue to carry the load. It can be seen from Figure 8 that although the *F* ^SBS^ of the untreated interleaved specimen (L2) was much lower than that of the all carbon-epoxy laminates (L1), plasma-treated samples (L3) showed an increase in *F* ^SBS^ compared to the untreated samples (L2). The results of short beam shear tests are listed in Table 4.

The increase in *F* ^SBS^ after plasma treatment indicated better adhesion characteristics between the DyneemaⓇ tape and the adjacent carbon-epoxy plies. It was also observed that the interleaved samples (L2 and L3) failed in a stepwise manner. For example, 50% of the L2 samples showed two load drops (dual-step failure) whereas only 25% of the L3 samples showed two load drops in the force vs. displacement curve before the maximum load (*P*_m_) was reached. These drops in the load were associated with the failure of either the DyneemaⓇ tape (L2 laminates) or the interface between Dyneema and adjacent carbon-epoxy plies (L3).

The load-carrying capacity of untreated BT10/carbon-epoxy samples (L2) was an exciting finding. It was expected that the ILSS in the case of the L2 specimen would be much lower than 26.0 ± 4.5 MPa (Table 4). For example, it was shown that the UHMWPE-epoxy composites had an ILSS of ≈1.7 MPa ^60^ and with a surface treatment such as atomic layer deposition (ADL), the ILSS increased to 2.5 MPa,^60^ which was far less in magnitude compared to 26.0 ± 4.5 MPa estimated in the present study. One reason for a significant difference could be a better methodology adopted in this study, which measured the intrinsic shear strength of the bonding of BT10 tape with adjacent CE/epoxy layers, whereas a 0°/90° configuration of DyneemaⓇ SK75 weave was tested, with five layers of UHMWPE fabric dipped in epoxy resin.

### 3.6 Failure of SBS Samples

The failure of the short-beam specimen is shown in Figure 9a–f. All samples were observed to fail by delamination. The applied load was taken up by the specimen until the load reached the strength of the weakest link. The weakest link in the case of interleaved samples could have been either the interface between DyneemaⓇ tape and the adjacent carbon-epoxy ply or the tape itself due to its low shear strength. Such a failure was observed as a drop in load in the force vs. displacement curves, where the load drop for untreated (L2) laminate was at significantly lower levels than the plasma treated (L3) laminates, i.e., 652.1 ± 124.7 N and 1066.3 ± 145.6 N, respectively. The attribution of failure to the tape or the interface was made based on the experimental observations and the physical state of samples after failure. The deformation and damage progression for sample L3 in Figure 9a–f showed that the ends of the specimen moved in opposite directions after delamination.

**Figure 9:**
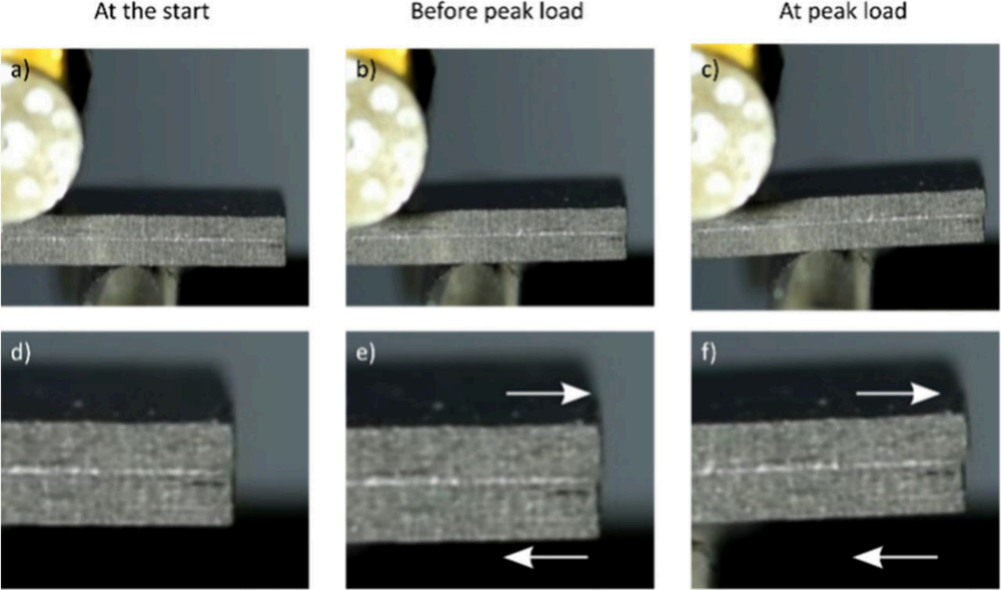
Deformation of short-beam shear specimen with carbon-epoxy plies and plasma-treated BT10 interleaf in the midsection of the specimen. The images show the state of samples at (a) the start of the test, (b) before reaching the peak load, and (c) at peak load. Similarly, the images in (d), (e), and (f) are the enlargements of images (a), (b), and (c), respectively. The samples were plasma-treated using Ar–O_2_ plasma at 100 W power, 50 mTorr process pressure, exposed for 10 s, where the gas flow rates of Ar and O_2_ were 8 SCCM and 42 SCCM, respectively.

Although it may be challenging to note the displacements of the top and bottom part of the specimen from the images in Figure 9a–f, it was evident from the testing videos that the failure occurred at the interface between DyneemaⓇ and carbon-epoxy plies for untreated (L2) laminates and within the DyneemaⓇ tape for plasma-treated (L3) laminates. The failed SBS specimens were analyzed using SEM to study the failure site. The SEM images of these specimens are shown in Figure 10a–d.

**Figure 10:**
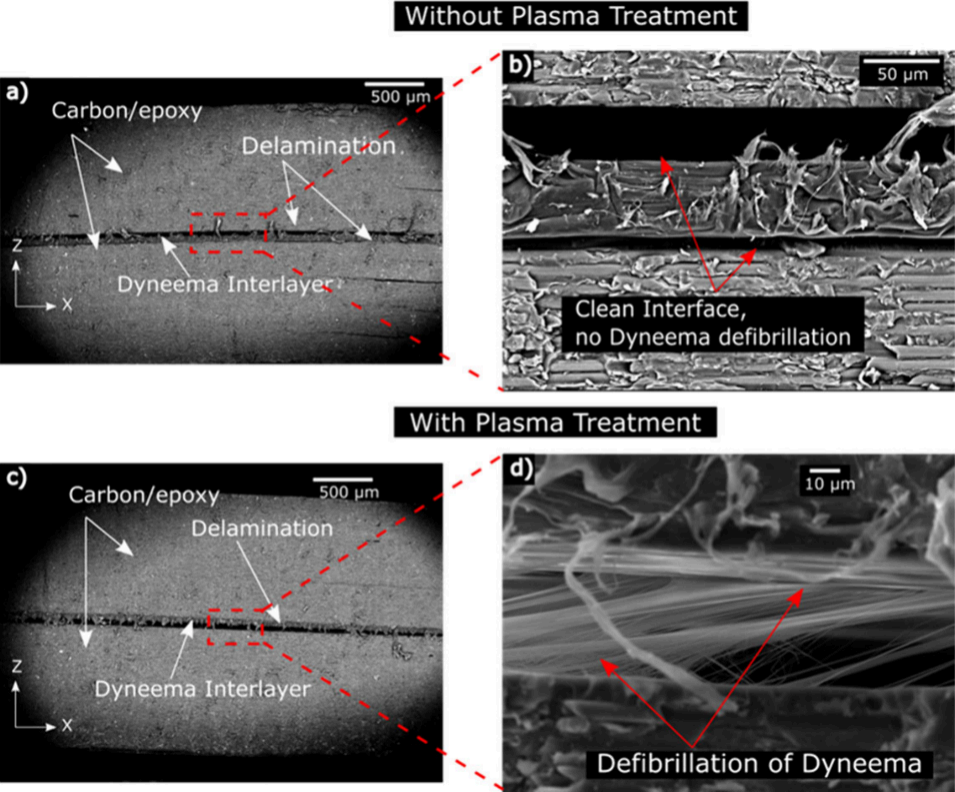
SEM images of SE84 LV laminates, sandwiching (a) untreated and (b) plasma-treated BT10 tape, exposed to plasma for 10 s. The high magnification images show (c) clean fracture for untreated tape and (d) defibrillation of tape, indicating higher adhesion between the adjacent carbon/epoxy plies.

The untreated BT10 tape was found to be intact with no defibrillation, indicating a low adhesion with the adjacent carbon-epoxy surfaces, as shown in Figure 10a,b. Such a failure was expected due to the nonpolar nature of the UHMWPE. However, the plasma-treated specimen showed that the tape underwent defibrillation, as shown in Figure 10c,d.

The defibrillation was attributed to the increased adhesion of the tape with the adjacent carbon-epoxy plies and the low shear strength of the tape itself, which was also suggested in another study.^61^ Thus, plasma treatment increased the interlaminar shear strength of the DyneemaⓇ BT10 tape carbon-epoxy composites, compared with the untreated material, along with a change in failure mode where DyneemaⓇ BT10 tape was observed to have initial bonding sites intact, contrary to the untreated samples which showed a clean fracture, as shown in Figure 10b.

The use of DyneemaⓇ fibers and tapes in structural composite applications has been limited due to the inherent inertness of the surface. The nonpolar surfaces of these fibers and tapes are not capable of interacting with the thermosetting matrices and thus a weaker interface is formed between the DyneemaⓇ reinforcement and the matrix.^62^ The present study has shown that the major limitation of the weak interface between DyneemaⓇ fibers and tapes and epoxy matrix can be overcome with the reactive ion etching (plasma) process, which significantly improves the adhesion at the micro and macro levels, as evident from the increased IFSS and ILSS values.

The increase in the adhesion was likely due to the addition of various oxygen-bearing chemical species after the plasma treatment, which promoted the interaction between the fiber and the matrix, leading to a stronger interface between the DyneemaⓇ tape and the epoxy matrix. Hence, after plasma treatment, the interface was no longer the weakest link and the failure of the specimen was then driven by the shear strength of the tape itself rather than the interface, i.e. cohesive failure as opposed to adhesive failure. The majority of the research work addresses the interface of DyneemaⓇ fibers and tapes, our findings suggest that the low shear properties of the fiber or tape will intrinsically limit applicability in a structural aspect where loads are applied off-axis, particularly inducing shear.

## 4 Conclusions

The Ar–O_2_ reactive ion etching enhanced the IFSS of Trevo90/Prime20TM LV system, from 2.1 to 5.1 MPa. The increase in IFSS was attributed to addition of oxygen-bearing functional groups (C–O–C, C=O, OH) because of plasma treatment. The plasma treatment was also observed to clean the surface of fiber, which could have led to a higher interaction between fiber and matrix, and hence an increase in IFSS.

The surface roughness *S*_q_ of the samples varied from 48 to 27 nm; however, no correlation between plasma parameters and *S*_q_ was observed. At lower exposure times (10 s), the surface roughness remained unchanged while IFSS increased significantly, compared to an untreated sample. At higher exposure times (60 and 300 s), *S*_q_ decreased significantly while IFSS plateaued at 5 MPa level. Thus, it was concluded that the surface roughness did not play a part in increased IFSS, and oxygen-bearing functionalities could be the main attributable cause for increased IFSS (2.1–5.9 MPa), as a result of plasma treatment.

The short beam shear tests showed a 63% increase in ILSS after 10 s of exposure to plasma, and SEM analysis of failed specimen showed a shift in failure mechanism from interfacial to cohesive, i.e., defibrillation of the tape rather than the failure of interface between fiber and matrix. Thus, plasma treatment effectively cleaned the surface of fiber and enhanced the fiber/matrix interaction at both micro- and macrolevels.

## Supplementary Material



## Notes

Figure S.1 showing representative SEM photomicrographs of untreated and plasma-treated Trevo90 fibers; Figure S.2 showing FTIR-ATR spectra and comparing functional groups on the surface of untreated and plasma-treated fibers; Figure S.3 showing relationship between embedded length of droplet and maximum force recorded during single fiber microbond test, comparing untreated and plasma-treated fibers; Figure S.4 showing droplet disbondment sites, in postpullout condition; Figure S.4 showing high-magnification image of debonded droplet of epoxy resin on single fiber, along with description of geometrical features of the microvise indentation; Figure S.5 showing the head and tail section of disbonded epoxy resin droplet on single fiber; Figure S.6 showing force vs displacement plots obtained through 3-point bend (short-bear shear), emphasizing the load drops prior to reaching point of maximum load (*P*_m_), comparing both untreated and plasma-treated BT10 interleaves; Table S.1 comparing surface roughness parameters obtained through HS-AFM experiments on single fibers in both untreated and plasma-treated states; Table S.2 showing assignment of FTIR spectral wavenumbers to chemical functional groups, complementing FTIR data in Figure S.2; Table S.3 showing load drop values for both single and dual type events, their magnitude, when samples with BT10 tape in untreated and plasma-treated conditions are subjected to short-bear shear tests (PDF)

## †

iAero Centre, Bunford Ln, Yeovil BA20 2EJ, United Kingdom
