# Supplementary material for: Improving Adhesion of UHMWPE with Epoxy Matrix by Reactive Ion Etching of UHMWPE Using Ar–O2 Plasma and the Effects of Plasma on Adhesion at the Micro- and Macroscale
Source: ACS Appl Mater Interfaces. 2025 Oct 9;17(42):58609–20. doi: 10.1021/acsami.5c10473 (PMC12557213; doi:10.1021/acsami.5c10473)
Supplement: Supplementary file 1 [file am5c10473_si_001.pdf]

SUPPORTING INFORMATION

Improving adhesion of UHMWPE with epoxy matrix by reactive ion etching of UHMWPE using Ar-O_2 plasma and the effects of plasma on adhesion at the micro and macro scale

Usman Sikander[a,1], Mark K. Hazzard[b], Ian Hamerton[a,*], Michael R. Wisnom[a]

[a]Bristol Composites Institute, School of Civil, Aerospace and Design Engineering, Faculty of Science and Engineering, Queen’s Building, University Walk, Bristol, BS8 1TR, United Kingdom
[b]DSM Materials Science Centre, Urmonderbaan 22, 6167 RD, Geleen, The Netherlands

1 Current address: iAero Centre, Bunford Ln, Yeovil BA20 2EJ, United Kingdom
* Corresponding Author, email address: ian.hamerton@bristol.ac.uk

Table of Contents

1 Supporting Figures 1

2 Supporting Tables 5

3 References 6

1 Supporting Figures

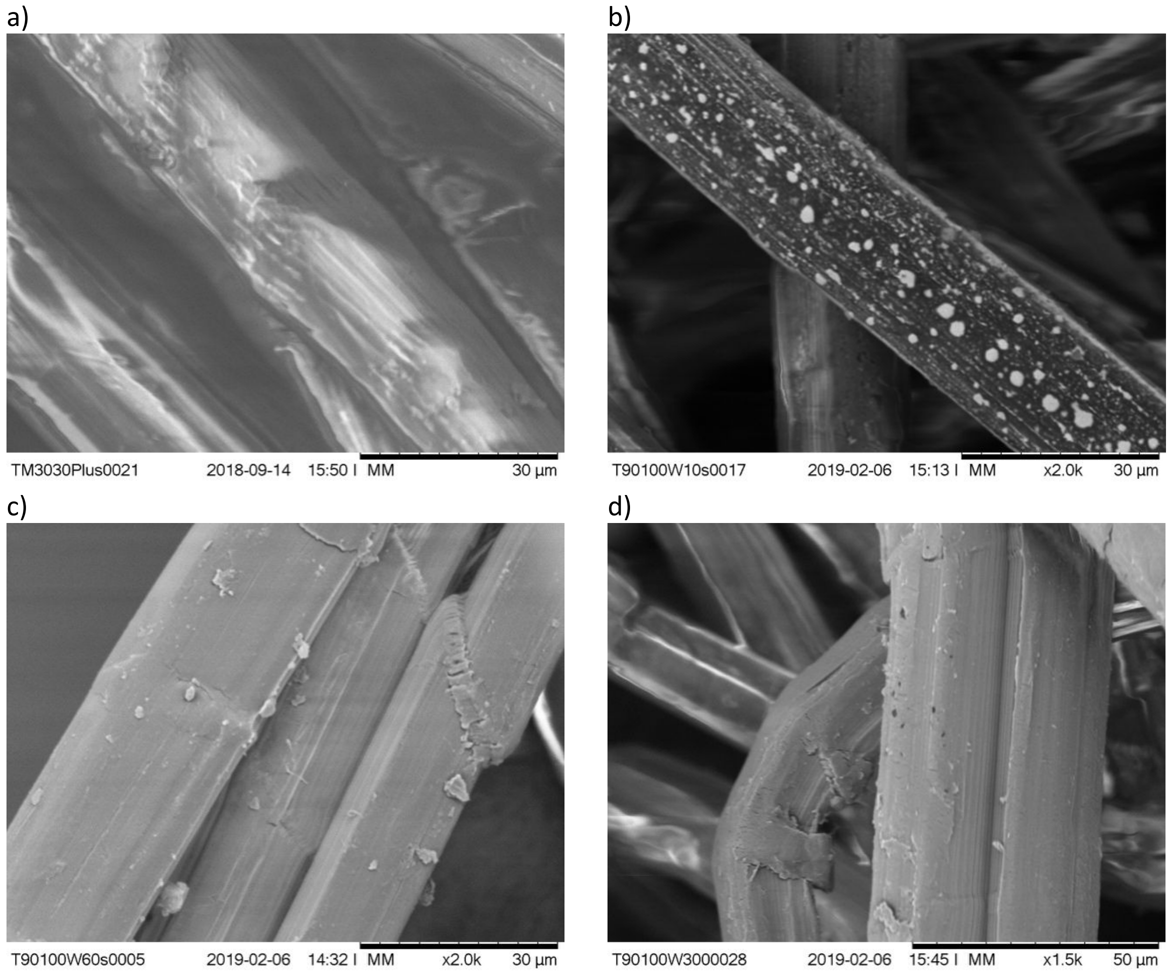

Figure S.1 Zoomed insets of SEM photomicrographs of Trevo 90 fibres in a) untreated and plasma-treated condition, exposed to plasma for b) 10 seconds, c) 60 seconds, and d) 300 seconds. Sample exposed to Ar-O_2 plasma at 100 RF power and 50 mTorr process pressure with gas flow rates of 42 and 8 SCCM for Ar and O_2, respectively.

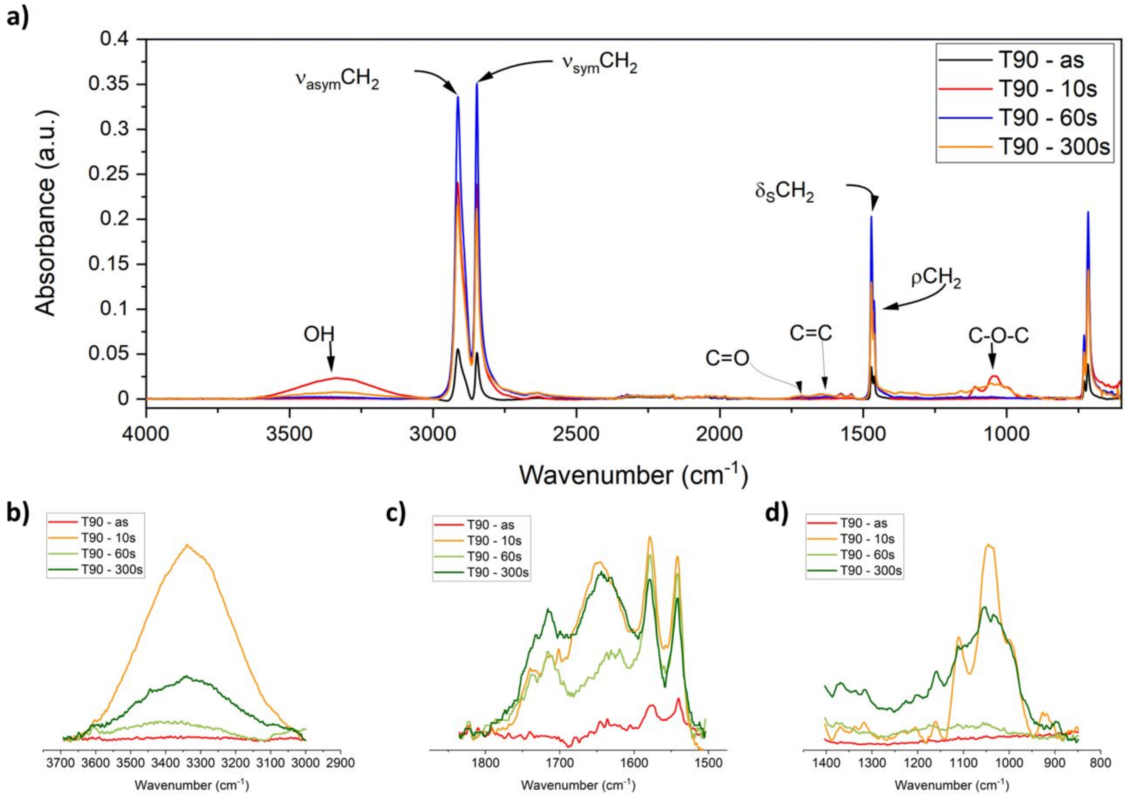

Figure S.2 FTIR spectra of a) untreated and plasma treated specimen, along with zoomed insets for wavenumber range of b) 3700 – 2900 cm^{-1}, c) 1800 – 1500 cm^{-1}, and d) 1400 – 800 cm^{-1}

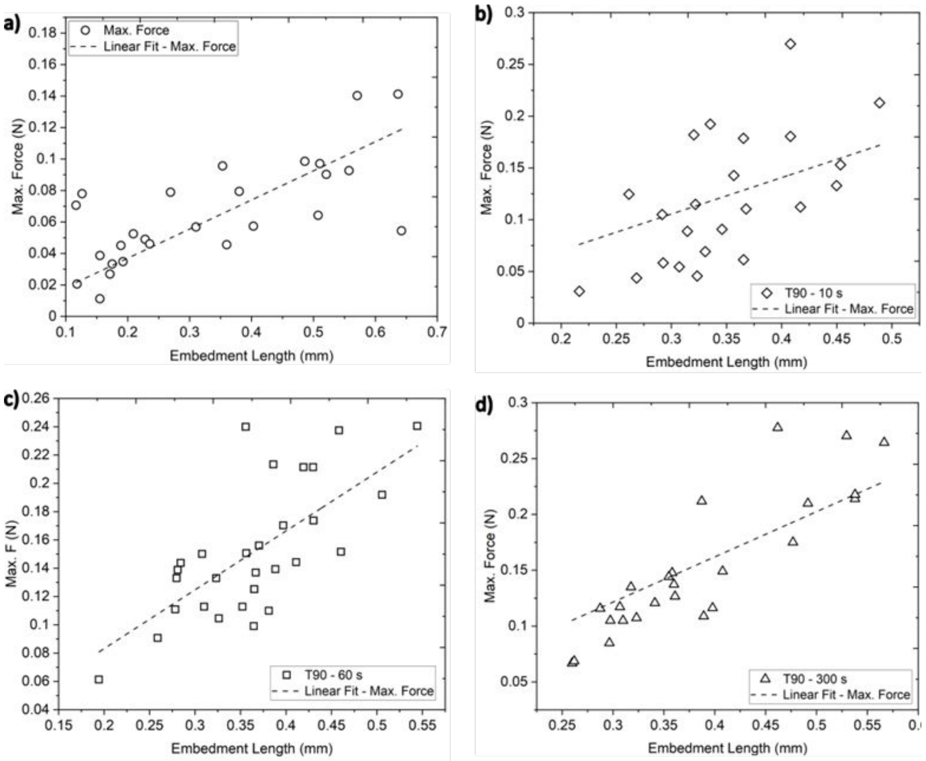

Figure S.3 Maximum force (F_{max}) *vs.* embedment length of microbond samples of (a) untreated and plasma-treated for (b) 10 seconds, (c) 60 seconds, and (d) 300 seconds.

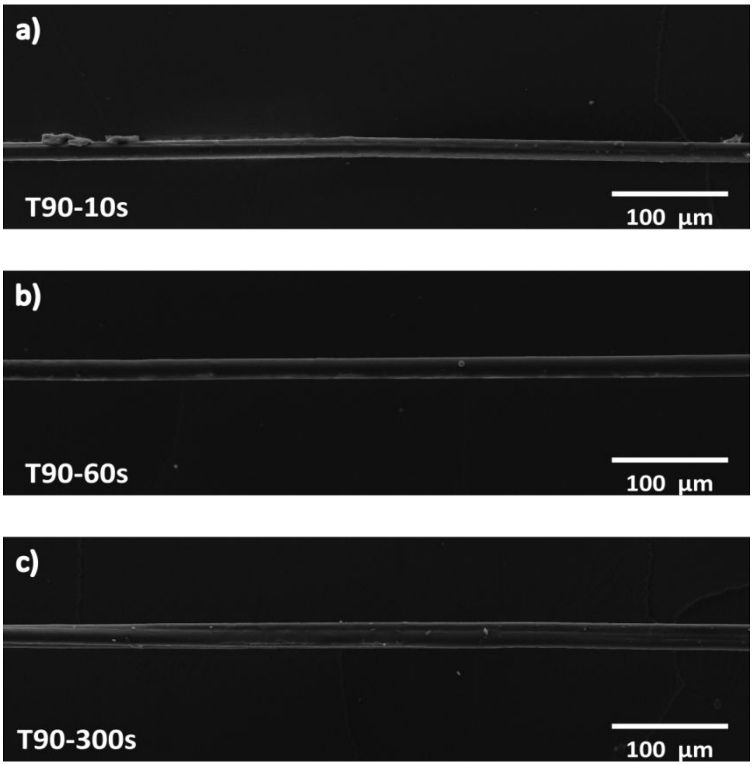

Figure S.4 Initial site of a droplet on a single fibre, after the droplets have been displaced on the fibres. The T90 fibres were plasma-treated using Ar-O_2 (1:5) at 100 W power, 50 mTorr process pressure for (a) 10, (b) 60, and (c) 300 seconds.

a)

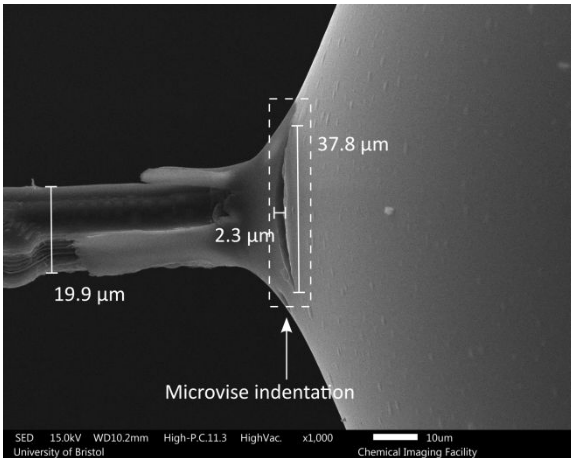

b)

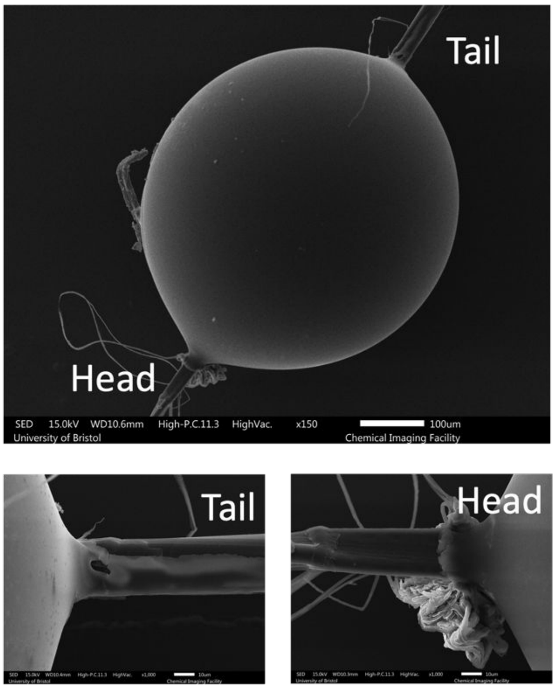

Figure S.5 SEM photomicrograph of failed microbond sample showing a) microvise indentation size, and b) the head and tail positions of the droplet.

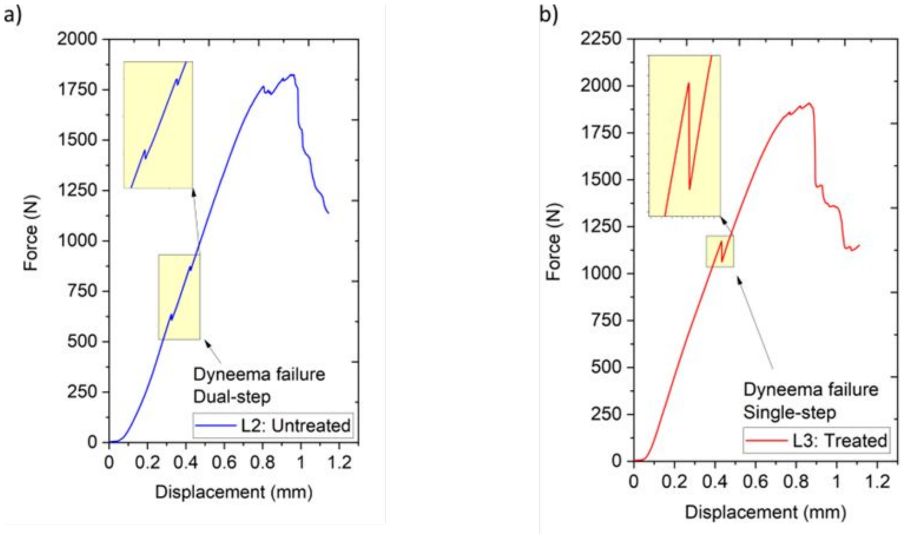

Figure S.6 Comparison of force *vs.* displacement curve of carbon-epoxy laminates interleaved with a) untreated BT10 tape and b) plasma-treated BT10 tape

2 Supporting Tables

Table S.1 Surface roughness measurements of Trevo90 fibres in an untreated and plasma-treated state. The table presents both the raw and flattened data

**Exposure** (s)	**Data type** (-)	$\mathbf{S_a}$ (nm)	$\mathbf{S_p}$ (nm)	$\mathbf{S_v}$ (nm)	$\mathbf{S_z}$ (nm)
0	Raw	37.5 ± 14.6	170.4 ± 80.4	169.9 ± 111.4	340.4 ± 177.5
10	Raw	41.3 ± 10.3	169.0 ± 37.8	205.6 ± 59.3	374.5 ± 87.9
60	Raw	10.1 ± 4.8	68.1 ± 21.4	41.8 ± 12.6	109.7 ± 33.0
300	Raw	21.5 ± 6.9	95.5 ± 20.7	85.6 ± 22.4	181.1 ± 31.3
0	Flat	1.4 ± 0.5	14.7 ± 9.2	13.6 ± 3.6	28.3 ± 9.9
10	Flat	1.8 ± 0.2	23.8 ± 8.8	15.7 ± 4.6	39.6 ± 10.1
60	Flat	1.7 ± 0.2	19.5 ± 3.5	14.5 ± 3.2	34.0 ± 4.7
300	Flat	1.6 ± 0.2	14.5 ± 3.1	11.6 ± 2.2	26.1 ± 4.9

Table S.2 FTIR spectral analysis of untreated and plasma-treated Trevo90 fibres

**Range** (cm^{-1})	**Species** (-)	**Vibration type** (-)	**Peak** (cm^{-1})	**Reference** (-)
3800-3000	Hydroxyl	v (OH)	3330	[1–3]
3050-2750	Methylene	v_{as} (CH_2)	2913	[4]
	Methylene	v_s (CH_2)	2847	[4]
1500-1400	Methylene	δ_s (CH_2)	1472	[4–6]
	Methylene	ρ (CH_2)	1462	[4–6]
1300-900	Anhydride	v_s (C-O)	1161	[7]
	Cyclic ether	v_s (C-O-C)	1111	[1,8]
	Ar-O-CH_2-O-Ar	v_s (=C-O)	1036	[1]
	Ether	v_s (C-O-C)	998	[8]
780-660	Methylene	δ (CH), out-of-plane	730	[1,2]
	Methylene	ρ (CH_2), in-phase	716	[4–6]

v_s: symmetric stretching vibration, v_{as}: asymmetric stretching vibration, δ_s: symmetric bending vibration, ρ: rocking vibrations

Table S.3 Comparison of untreated and plasma-treated samples in terms of start, end and magnitude of load drop at the peak loads (P_m) for L2 and L3 laminates

**Laminate** (-)	**Type** (-)	**Load drop: start** (N)	**Load drop: end** (N)	**Load Drop** (N)
2	Single	736.3 ± 146.2	665.5 ± 142.1	-70.8 ± 10.4
2	Dual	667.3 ± 138.6	644.6 ± 134.1	-22.6 ± 7.3
3	Single	1104.8 ± 82.6	1023.3 ± 62.9	-75.4 ± 40.5
3	Dual	606.2 ± 144.5	588.0 ± 137.1	-20.0 ± 16.7

3 References

(1) Socrates, G. *Infrared and Raman Characteristic Group Frequencies. Tables and Charts*; 2001.

(2) Coates, J. *Interpretation of Infrared Spectra, a Practical Approach*; 2000.

(3) Geyter, N. D.; Morent, R.; Leys, C. Surface Characterization of Plasma-Modified Polyethylene by Contact Angle Experiments and ATR-FTIR Spectroscopy. In *Surface and Interface Analysis*; 2008; Vol. 40, pp 608–611. https://doi.org/10.1002/sia.2611.

(4) Li, W.; Ma, R.; Huang, M.; Meng, L.; Pan, Q. Surface Treatment of Ultra-High Molecular Weight Polyethylene Fibers Using Potassium Permanganate and Mechanical Properties of Its Composites. *Surf. Interface Anal.* **2018**, *50* (1), 65–72. https://doi.org/10.1002/sia.6336.

(5) Li, W.; Huang, M.; Ma, R. Improved Mechanical Properties of Epoxy Composites Reinforced with Surface-Treated UHMWPE Fibers. *Polym. Adv. Technol.* **2018**, *29* (4), 1287–1293. https://doi.org/10.1002/pat.4240.

(6) Silverstein, R. W.; Bassler, G. C. Spectrometric Identification of Organic Compounds. *J. Chem. Educ.* **1962**, *39* (11), 546–553. https://doi.org/10.1021/ed039p546.

(7) Stuart, B. H. *Infrared Spectroscopy: Fundamentals and Applications*; 2005. https://doi.org/10.1002/0470011149.

(8) Fejdyś, M.; Łandwijt, M.; Kucharska-Jastrzabek, A.; Struszczyk, M. H. The Effect of Processing Conditions on the Performance of UHMWPE-Fibre Reinforced Polymer Matrix Composites. *Fibres Text. East. Eur.* **2016**, *24* (4), 112–120. https://doi.org/10.5604/12303666.1201140.
